# PEG-Lipids: Quantitative Study of Unimers and Aggregates Thereof by the Methods of Molecular Hydrodynamics

**DOI:** 10.1021/acs.analchem.3c01999

**Published:** 2023-07-07

**Authors:** Ilya Anufriev, Stephanie Hoeppener, Ivo Nischang

**Affiliations:** † Laboratory of Organic and Macromolecular Chemistry (IOMC), Friedrich Schiller University Jena, Humboldtstraße 10, 07743 Jena, Germany; ‡ Jena Center for Soft Matter, Friedrich Schiller University Jena, Philosophenweg 7, 07743 Jena, Germany

## Abstract

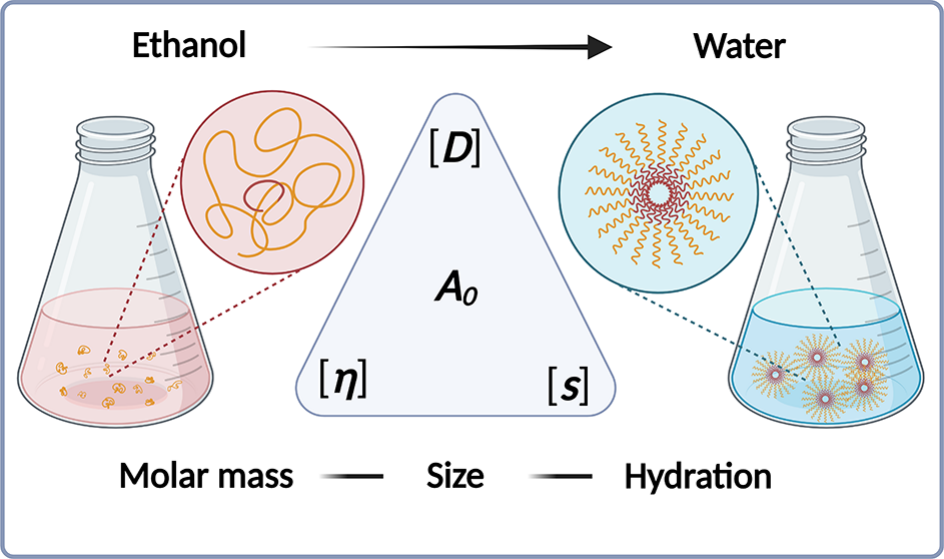

Understanding the polymorphism of lipids in solution is the key to the development of intracellular delivery systems. Here, we study the dynamics of poly(ethylene glycol)-lipid (PEG-Lipid) conjugates aiming at a better understanding of their molecular properties and aggregation behavior in solution. Those PEG-Lipids are used as components of lipid nanoparticles (LNPs). LNPs are gaining increased popularity, e.g., by their utilization in modern vaccination strategies against SARS-CoV-2. Characterization of the systems is conducted by the classical methods of hydrodynamics in different solvents, such as ethanol and water, which are also commonly used for LNP formulation. We were able to elucidate the structurally associated hydrodynamic properties of isolated PEG-Lipids in ethanol, revealing the typically expected values of the hydrodynamic invariant for random coil polymers. By virtue of the same experimental setting, the PEG-Lipids’ behavior in water was as well studied, which is a less good solvent than ethanol for the PEG-Lipids. Our experiments demonstrate that PEG-Lipids dissolved in water form well-defined micelles that can quantitatively be characterized in terms of their degree of aggregation of PEG-Lipid polymer unimers, their hydrodynamic size, and solvation, i.e., the quantitative determination of water contained or associated to the identified micelles. Quantitative results obtained from classical hydrodynamic analyses are fully supported by studies with standard dynamic light scattering (DLS). The obtained diffusion coefficients and hydrodynamic sizes are in excellent agreement with numerical results derived from analytical ultracentrifugation (AUC) data. Cryo-transmission electron microscopy (cryo-TEM) supports the structural insight from hydrodynamic studies, particularly, in terms of the observed spherical structure of the formed micelles. We demonstrate experimentally that the micelle systems can be considered as solvent-permeable, hydrated spheres.

## Introduction

**Scheme 1:**
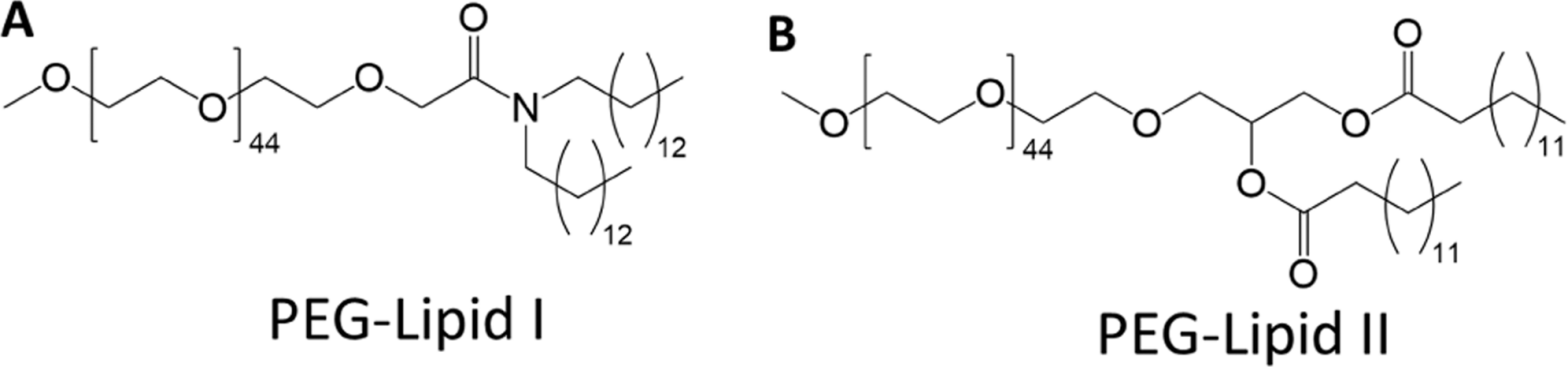
Structural Formulas of the PEG-Lipids: (A) PEG-Lipid I and (B) PEG-Lipid II

The outstanding role of poly(ethylene glycol)-lipids (PEG-Lipids) as pharmapolymers is reflected in their utilization, e.g., for the formulation of mRNA vaccines.^1^ The respective PEG-Lipids play an important role in formulation strategies and are characterized by a PEG backbone bearing a lipid terminus (PEG-Lipids I and II) (Scheme 1). These PEG-Lipids are particularly interesting, since they are used for the commercial production and distribution of the most successful mRNA vaccines. However, it is suspected that the wide use of PEGs in our modern society^2^ is associated with undesired side effects by vaccination, the origins of these being still under debate.^3^ Next to the pharmapolymer PEG with stealth properties, alternatives have been proposed and quantitatively studied.^4,5^ These include water-soluble linear pharmapolymers such as, e.g., poly(2-alkyl-2-oxazoline)s (POxs),^6,7^ linear poly(glycerol)s (LPGs),^5,8,9^ and hyperbranched poly(glycerol)s (HPGs),^10^ to mention just a few.

PEGs have been studied extensively with respect to their molecular hydrodynamic properties and conformation in solution.^11^ Here, in particular, the interrelation of hydrodynamic data is essential for a deeper understanding of the polymers.^7,11,12^

Next to hydrodynamic characterization of PEGs themselves, also conjugates of PEG with biological materials have received attention.^13^ The physicochemical properties of PEG conjugates were elucidated by experimental methods of hydrodynamics, particularly in terms of the hydrodynamic properties of the conjugate.^8,14−16^ Also, studies of PEG conjugates with lipids^17^ or mRNA-lipid nanoparticles (LNPs) containing PEG^18^ have been reported, including the use of analytical ultracentrifugation (AUC).

The quantitative study of polymer systems is a challenging task and typically cannot rely on a single analytical approach but requires a combination of orthogonal and complementary methods, addressing a variety of aspects of the materials in solution. A key technological platform for the absolute study of polymer systems is an analytical ultracentrifuge that found its inception with Svedberg’s pioneering work on disperse systems.^19,20^ In combination with viscometry of macromolecular systems, first proposed by Staudinger,^21−24^ the methods of macromolecular hydrodynamics have developed into powerful approaches in the study of polymers of natural and synthetic origin.^12^ The peculiar advantage of AUC emanates from the study of materials in solution under action of a centrifugal force in the hermetically closed system of conserved mass balance.^25−27^ The materials are investigated by suitable detectors in solution without global dilution and absence of sample processing issues.

The in situ observed transport of material in the cell sectors for AUC experiments allows for extraction of primary hydrodynamic data, particularly sedimentation and diffusion coefficients, associated to the materials concentration.^28^ In combination with primary data on molecular density and viscometric properties, it can decipher macromolecular and colloidal parameters, ultimately determining the molar masses of the materials in solution.^10,29,30^

Herein, we study the solution properties of PEG-Lipids I and II (Scheme 1) in terms of the methods of molecular hydrodynamics involving (molecular) viscometry, (molecular) densimetry, and sedimentation–diffusion analysis making use of AUC. These experiments were performed to estimate both translational diffusion coefficients and sedimentation coefficients of the materials in the solvents ethanol and water. The properties of the systems formed in water, next to the previously mentioned quantitative hydrodynamic analysis, were additionally studied by dynamic light scattering (DLS) and cryo-transmission electron microscopy (cryo-TEM). In a self-sufficient hydrodynamic approach through intrinsic property estimation, the materials were studied concerning their degree of aggregation in water emanating from the PEG-Lipid molecular entity, their hydrodynamic invariant, and their level of hydration based on translational and rotational friction data. Ultimately, an enhanced understanding of the hydrodynamic characteristics of PEG-Lipid conjugates in solution can substantiate progress in attempts of their replacement in nanomedicine, where quantitative structure–property relationships are needed.

## Theoretical Background

### Diffusion and Sedimentation

Combining considerations of thermodynamics and hydrodynamics, the classical Stokes–Einstein equation for diffusion, *D*, reads^31^
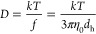
1where *k* is the Boltzmann constant, *T* is the absolute temperature, and *f* is the translational friction coefficient of the species under consideration. For a solid sphere *f* = 3π*η*_0_*d*_h_, with *η*_0_ being the solvent viscosity. The above equation builds the basis for the calculation of hydrodynamic diameters, *d*_h_, from diffusion coefficients, *D*. Those *d*_h_values refer to the size of an ideal solid sphere, diffusing at the same speed as the objects under experimental observation. Perhaps the most common way is the determination of *d*_*h*_values by dynamic light scattering (DLS) experiments. DLS originally assesses translational diffusion coefficients, *D*.^26,32,33^

Another attractive opportunity to determine *D* is the use of sedimentation velocity experiments performed in AUC and numerical solution of the Lamm equation providing access to both sedimentation coefficients, *s*, and translational diffusion coefficients, *D*, under the assumption of a single population of species of certain concentration, *c*(34,35)
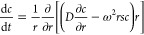
2The numerical solution of the above equation, radial- and time-resolved, estimates *s* in a given centrifugal field, ω^2^*r*, and *D* due to developed concentration gradients, ∂*c*/∂*r*, in the typically sector-shaped cells.^34,35^*D*, under conditions of sedimentation, is defined by
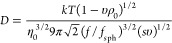
3We note, that *D* can be expressed in terms of a translational frictional ratio, *f*/*f*_sph_, with *f* being the translational friction coefficient of the species and *f*_sph_ that of a spherical particle of the same anhydrous volume and mass.^7,11^*υ* is the partial specific volume of the species and *ρ*_0_ is the solvent density.

### Intrinsic System Properties

Based on determined sedimentation coefficients, *s*, from objects in solution with a particular partial specific volume, *υ*, in solvents of precisely known viscosity, *η*_0_, and density, *ρ*_0_, the intrinsic sedimentation coefficient [*s*] is defined by
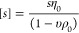
4where (1 – *υρ*_0_) is the buoyancy factor.

The intrinsic diffusion coefficient, [*D*], of those objects is defined by
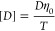
5

Intrinsic estimates are particularly useful, because they allow for solution system comparability, i.e., when experiments in differently dense and viscous solvents are performed.^25,26,36^

It appears useful here to also introduce the concept of the intrinsic viscosity, [*η*], a third hydrodynamic characteristic^21^
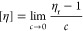
6where *η*_r_ is the relative viscosity of the polymer or colloid solution. Classically, [*η*] values are determined by the Huggins^37^ and Kraemer^38^ extrapolation procedures in a typical range of relative viscosities, 1.2 ≤ *η*_r_ ≤ 2.5, that should result in linear plots
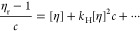
7
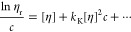
8Next to [*η*] other associated hydrodynamic characteristics are the Huggins, *k*_H_, and Kraemer parameters, *k*_K_, indicating the quality of the solvent for a particular polymer solvent system.

The interplay between the intrinsic characteristics, [*η*] (in numerical values *dLg*^–1^), [*s*], and [*D*], is commonly established with the hydrodynamic invariant, *A*_0_, representing a statistical average and physically meaningful range of values for polymer and colloidal systems^12,39,40^
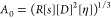
9where *R* is the universal gas constant.

## Governing Relations for Molar Mass, Size, and Hydration

### Molar Mass

The Svedberg equation for molar mass estimations, together with the above relations, can take the following forms^10,29,30^
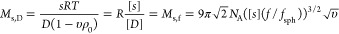
10where *M*_s,D_ is the molar mass based on intrinsic sedimentation, [*s*] (eq 4), and intrinsic diffusion coefficients, [*D*] (eq 5). *N*_A_ is the Avogadro number. In a modified form, *M*_s,f_ is the molar mass based on translational frictional ratios, *f*/*f*_sph_ (eq 3), and intrinsic sedimentation coefficients, [*s*] (eq 4).

### Hydrodynamic Diameter

In analogy to the above considerations, hydrodynamic diameters can be calculated and derived by substituting *D* in eq 1 with the expression for *D* from eq 3 derived from AUC data and rearranging for *d*_h_.^28^ Together with the definition of [*s*] (eq 4), we obtain the following expression for hydrodynamic diameters, *d*_h_, from sedimentation velocity experiments in AUC
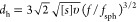
11For an ideal solid sphere, *f*/*f*_sph_ values are obsolete in eq 11 (i.e., *f*/*f*_sph_ = 1) and *d*_h_values are those of a spherical particle sedimenting at the same speed as the objects under observation.^26^ In the literature, those are referred to as a sedimentation-equivalent hydrodynamic diameter since altered translational friction properties, due to hydration and/or shape anisotropy, are neglected.^26,41,42^

### Hydration

At this point, it is worthwhile to take a closer look at *f*/*f*_sph_values, which are often referred to describe shape anisotropy, i.e., by the classical Perrin friction factor, *P*.^43^ However, regarding the interpretation of *f*/*f*_sph_ from sedimentation–diffusion analysis, one must consider hydration, *δ*_AUC_, of the objects under investigation^10,44^
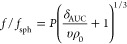
12which simplifies for a spherical shape (*P* = 1). In this case, hydration may straightforwardly be interpreted by the translational friction properties only
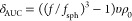
13Another important metric allowing to understand hydration is the intrinsic viscosity, [*η*] (eq 6), which is related to the specific volume, *V*_s_, and the molar mass, *M*. It can be defined as^45^
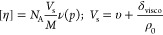
14where *V*_s_ is the specific volume of the macromolecules in solutions, *ν*(*p*) is the dimensionless viscosity increment, and *δ*_visco_ is the hydration of the macromolecules or objects in solution. The dimensionless viscosity increment, also referred to as the so-called Simha function, ν(*p*), will be equal to a value of 2.5 for spherical anhydrous particles.^46^ In this case, Einstein’s relation, i.e., [*η*] = 2.5*υ*, is obtained.^10,47^Equation 14 leads one to the following relationship for the value of hydration
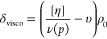
15

## Experimental Section

### Materials

The PEG-Lipids I (α-[2-(ditetradecylamino)-2-oxoethyl]-omega-methoxy-poly(oxy-1,2-ethanediyl), ALC-0159) and II (1,2-dimyristoyl-rac-glycero-3-methoxypolyethylene glycol-2000, DMG-PEG 2000) were purchased from Biomol (Hamburg, Germany) and Sigma-Aldrich (now Merck, Darmstadt, Germany) (Scheme 1). Ethanol was obtained from Merck (Darmstadt, Germany). Ultrapure water was received from a Thermo Scientific Barnstead GenPure xCAD Plus water purification system (Thermo Electron LED GmbH, Langenselbold, Germany). PEG-Lipid I (ALC-0159), by visual inspection, dissolved in both solvents without heating within 30 min. PEG-lipid II (DMG-PEG 2000) dissolved only by heating in both solvents up to 50 °C with a total dissolution time of 1 h.

### Viscometry

For the experiments and evaluation of the intrinsic viscosity, [*η*], solution viscosity measurements at varying concentrations of material in solvents ethanol and water were performed using a Lovis 2000ME Microviscometer (Anton Paar, Graz, Austria) based on the moving ball principle. The experiments were performed in analogy to recent work.^7,11^ Therefore, the respective ball times in solvent, *t*_0_, and in the polymer solutions of particular concentrations, *t*_c_, were obtained using a capillary (made of glass with an inner diameter of 1.59 mm and a length of 25 mm) at PEG-Lipid concentrations where relative viscosities, *η*_r_ = *t*_c_/*t*_0_, were in an approximate range of 1.2 ≤ *η*_r_ ≤ 2.5. Experiments were conducted at a capillary–ball inclination angle of 50° and at a temperature of *T* = 20 °C.

### Densimetry

The density measurements were performed using a densimeter DMA 4500M (Anton Paar, Graz, Austria) according to the procedure described in previous works.^7,11^ Therefore, the PEG-Lipids were prepared in a concentration range of 0.2–1.0 × 10^–3^ g cm^*–*3^ and the solution densities, *ρ*_c_, were measured. Then, the density increment, by subtracting the solvent density, *ρ*_0_, from *ρ*_c_, (*ρ*_c_ – *ρ*_0_), was plotted against the mass concentration. The linear slope of the density increments, representing the buoyancy factor, (1 – *υρ*_0_), was used to calculate the partial specific volume, *υ*, of the PEG-Lipids. All experiments were performed at a temperature of *T* = 20 °C.

### Analytical Ultracentrifugation (AUC) Experiments

Sedimentation velocity experiments were carried out with a ProteomeLab XL-I analytical ultracentrifuge (Beckman Coulter, Brea, CA), using double-sector Epon (for hydrodynamic studies in water) or aluminum (for studies in ethanol) centerpieces with a 12 mm optical solution path length assembled in the AUC cells. The cells were placed in an eight-hole rotor (An-50Ti). The sector-shaped reservoirs within the centerpieces contained in the cells were filled with ca. 440 μL of pure solvent (water or ethanol) in the reference sector and ca. 420 μL of the PEG-Lipid solutions in the sample sector. Before AUC runs, the cell-loaded rotor was equilibrated for 1 h at a temperature of *T* = 20 °C in the centrifuge chamber. Sedimentation velocity runs, at a rotor speed of 42 000 rpm, were conducted at a temperature of *T* = 20 °C for an overall of 48 h. The radial- and time-resolved sedimentation velocity profiles were recorded by the interference optical detection system at a time interval of 3 min. A suitable selection of scans was used for numerical analysis. For experiments in water, the repeatability of experiments was checked for by manually shaking the cells at random times within 15 min after the AUC run. Then, the sedimentation velocity experiments with identical cells and their contained, presumably reconstituted, solutions were repeated. This was followed by the same experimental and numerical analysis procedure.

### Dynamic Light Scattering (DLS)

The diffusion coefficients, *D*, and *z*-average hydrodynamic diameters, *d*_h, DLS_, of the studied samples in water were determined by using a Zetasizer Nano-ZS (Malvern Instruments Ltd., Worcestershire, U.K.). The instrument is equipped with a 663 nm He–Ne laser. Intensity fluctuations were measured at a backscattering angle of 175° and at a temperature of *T* = 20 °C. The *d*_h, DLS_ is obtained by cumulant analysis of the decay functions and allows estimation of diffusion coefficients, *D*, and consequently hydrodynamic diameters, *d*_h, DLS_ (eq 1). The latter are accompanied with a polydispersity index (PDI), setting into relation the standard deviation of the investigated population and the hydrodynamic diameters.^26^

### Cryo-Transmission Electron Microscopy (Cryo-TEM)

Cryo-TEM investigations were conducted on a FEI Tecnai G^2^ 20 with an acceleration voltage of 200 kV. Samples (9 μL of 10 mg mL^–1^ concentrated PEG-Lipid) were applied onto hydrophilized Quantifoil grids (Quantifoil, Germany, R2/2) utilizing a Vitrobot Mark IV vitrification system and were transferred to the cryo-TEM holder (Gatan) utilizing a Gatan cryostage, maintaining always a temperature below −175 °C. Images were acquired on a 1 × 1k or a 4 × 4k CCD camera.

## Results and Discussion

### Intrinsic Viscosity

**Figure 1:**
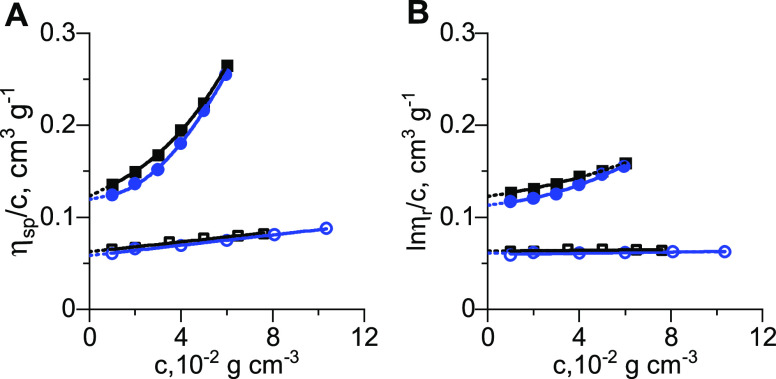
(A) Huggins and (B) Kraemer extrapolation plots (eqs 6–8) to estimate intrinsic viscosities, [*η*], of PEG-Lipids I (black color) and II (blue color) in solvents ethanol (open symbols) and water (closed symbols). Note that for the extrapolation plots in water, a quadratic and cubic extension of concentration, *c*, for the determination of [*η*] (eq 7) was used such that classical Huggins, *k*_H_, and Kraemer, *k*_K_, parameters in Table S1 are not available.

Figure 1 shows Huggins and Kraemer plots of the PEG-Lipids (Scheme 1) in the solvents ethanol and water. In ethanol, the plots display the typical linear relations resulting in estimates of the intrinsic viscosity, [*η*], in a range of 6–7 cm^3^ g^–1^ (eqs 6–8 and Table S1), as expected for the relatively small molar masses of polymers.^7,11^ The obtained values suggest molecularly dissolved coiled unimers of the PEG-Lipids. The corresponding Huggins, *k*_H_, and Kraemer, *k*_K_, parameters in the solvent ethanol (Table S1) indicate poor solvent quality, which appears reasonable based on the lipophilic terminal group of the small molar mass PEG polymers (Scheme 1).^11^

When utilizing water as a solvent, it is apparent that the classical extrapolation plots result in nonlinear dependencies within the typical test range of relative viscosities, particularly for the Huggins plots (Figure 1). To account for this nonlinear dependence, we fitted a power function to determine [*η*] values, i.e., extending eqs 7 and 8 by the term *k*[*η*]^3^ *c*^2^ + *k*[*η*]^4^ *c*^3^ (a quadratic extension for the concentration, *c*, by itself was not sufficient to significantly increase the fit quality). The apparent and unusual nonlinearity of the Huggins and Kraemer plots, requiring term expansion as opposed to typical polymer systems within the range of relative viscosities investigated here, could be explained by dynamic aspects of the highly hydrated noncovalently formed micelle system (vide infra). In this way, both extrapolation procedures (Huggins and Kraemer) lead to very similar values of the intrinsic viscosity [*η*]. The obtained values of [*η*] (∼12 cm^3^ g^–1^) are significantly larger in water than in ethanol (Table S1), which, at first sight, indicates structural changes of the polymer system of yet unknown origin (vide infra).

### Partial Specific Volume

The partial specific volume, *υ*, was determined by density increment measurements (Figure S1). The results indicate higher values of *υ* when compared to typical values for PEG analogues (*υ* = 0.83 cm^3^ g^–1^),^11^ however, being very similar in solvents ethanol (*υ* = 0.91 cm^3^ g^–1^) and water (*υ* = 0.89 cm^3^ g^–1^) for both PEG-Lipids (Table S1). Clearly, the lipid terminus significantly influences the partial specific volume, *υ*, of the PEG-Lipid conjugates, ultimately influencing their hydrodynamic properties in AUC experiments and being critical for sedimentation–diffusion analysis (eqs 2 and 3).

### Sedimentation Velocity AUC Experiments

**Figure 2:**
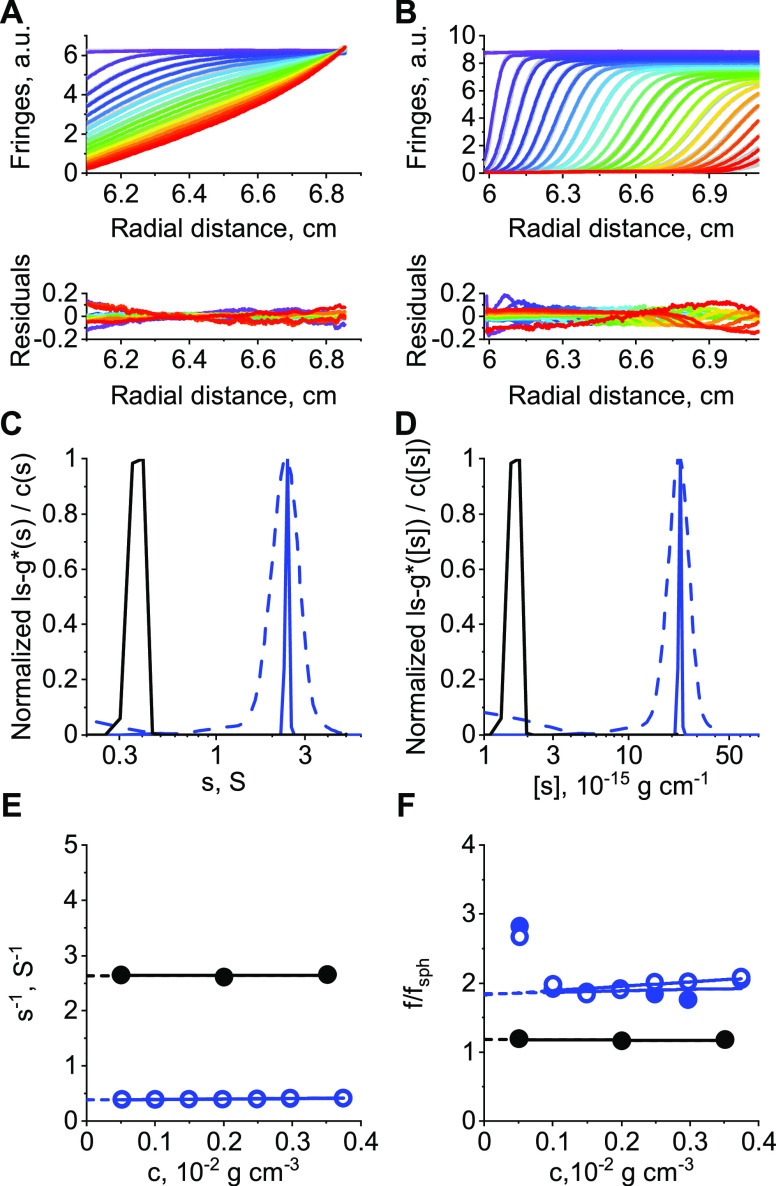
Time-resolved radial distance scans (gray symbols) and numerical results from sedimentation–diffusion analysis, *c*(*s*), (colored lines) of PEG-Lipid I in (A) ethanol and (B) water at a concentration of *c* = 2 mg mL^–1^. (C) Differential distributions of sedimentation coefficients from sedimentation–diffusion analysis, *c*(*s*) (solid black line in solvent ethanol, solid blue line in solvent water), and without considering effects of diffusion, *ls* – g*(*s*) (dashed blue line in solvent water). (D) Differential distributions of intrinsic sedimentation coefficients, *c*([*s*]) and *ls* – g*([*s*]), based on panel (C) and derived on the basis of eq 4. Concentration dependence of (E) signal (weight) average inverse sedimentation coefficients, *s*, and (F) weight-average translational frictional ratios, *f*/*f*_sph_. Black symbols and lines in panels (E) and (F) refer to solvent ethanol with averages shown. Blue symbols and lines refer to solvent water with linear fits and extrapolation to infinite dilution. Open blue symbols refer to stability experiments.

Figure 2 shows the results from AUC experiments for PEG-Lipid I. The results for PEG-Lipid II are presented in Figure S2. Figures 2A and S2A show time-resolved radial distance scans as observed in AUC and results from modeling in solvent ethanol and Figures 2B and S2B in solvent water. It is clear that the material sediments faster in water than in ethanol. Differential distributions of sedimentation coefficients, *c*(*s*),^34^ highlight this difference as seen in Figures 2C and S2C and which is as well seen in Table S1. Next to sedimentation–diffusion analysis, the sedimentation velocity data in water were also modeled without considering effects of diffusion, i.e., by least-squares boundary analysis, *ls* – g*(*s*) (see dotted lines in Figures 2C and S2C).^48^ Both modeling approaches led to coinciding signal (weight) average sedimentation coefficients such that the one from the *c*(*s*) analysis was utilized (Table S1).

To account for differences in solvent properties in terms of viscosity and density, we established an intrinsic sedimentation scale, *c*([*s*]), based on eq 4, allowing for a quantitative comparison.^36^ It is clear that the material sedimenting in water shows an order of magnitude larger intrinsic sedimentation coefficients, [*s*], than the very same material in ethanol (see Figures 2D and S2D and Table S1). This clearly suggests structural changes in the polymer system in an absolute fashion.

Concentration-dependent investigations were performed at high degrees of dilution, characterized by the product of mass concentration and intrinsic viscosity, *c*[*η*].^29,30^*c*[*η*] varied in the range of 3.0 × 10^–3^ ≤ *c*[*η*] ≤ 2.0 × 10^–2^ in ethanol and 4.0 × 10^–3^ ≤ *c*[*η*] ≤ 5.0 × 10^–2^ in water. Such degrees of dilution mean that the materials occupy less than 5% of the volume of the solution, even at the highest concentration investigated here. Investigations in ethanol at different concentrations indicate an apparent absence of a concentration dependence of the inverse sedimentation coefficients, *s*^–1^, (Figures 2E and S2E) and translational frictional ratios, *f*/*f*_sph_ (Figures 2F and S2F). This means that no nonideality can be resolved at the here used high degrees of dilutions (vide supra). Therefore, for all calculations, average values and numerical results from all concentrations in ethanol were utilized. For the materials investigated in water, a slight dependence of inverse sedimentation coefficients, *s*^–1^, (Figures 2E and S2E) and an unusual but repeatable dependence of translational frictional ratios, *f*/*f*_sph_, are apparent (Figures 2F and S2F). For water, linear fits to those data were utilized to identify values at infinite dilution.^11^ For the lowest concentrations in water, we noted poorer convergence of the model to the actual sedimentation velocity data (Figures 2F, S2F, and S3). Numerical analysis of the data (Figure S3, colored lines) indicates substantial deviation of model profiles from the original data, particularly observable at the upper and lower ranges of the boundaries (Figure S3, gray symbols). This means that the average *f*/*f*_sph_ values from the model are different from what the data may suggest. Therefore, *f*/*f*_sph_ values at the lowest investigated concentration in water were not considered for fitting of data in Figures 2F and S2F.

Numerical solution of the Lamm equation (eq 2) by sedimentation–diffusion analysis, *c*(*s*),^11,34^ results in molar masses (right hand side of eq 10) of the species in ethanol of *M*_s,f_ = 2000 g mol^–1^ for PEG-Lipid I and *M*_s,f_ = 2100 g mol^–1^ for PEG-Lipid II (when compared to their values in water (*M*_s,f_ = 224 000 g mol^–1^ for PEG-Lipid I and *M*_s,f_ = 269 000 g mol^–1^ for PEG-Lipid II, Table S1)). Apparently, the molar masses estimated in water are two orders of magnitude larger than in ethanol. Also striking are the much larger *f*/*f*_sph_ values for the materials in water (Figures 2F and S2F and Table S1). The molar mass estimations (Table S1) suggest an aggregation number, *N*_agg_, of about 120 individual PEG-Lipid polymer chains (Table S2).

Finally, a simple experiment to assess the colloidal stability of the materials in water was performed by briefly shaking of the centrifuge cells after centrifugation for 48 h at 42 000 rpm. The experiments demonstrated full redispersibility and provided exactly the same result from repeated sedimentation–diffusion analysis in terms of all determined hydrodynamic characteristics (see dashed versus solid lines in Figure S4A,B for differential distributions of sedimentation coefficients, *c*(*s*), from experimental data as well as open and closed symbols from modeling in Figures 2E,F and S2E,F).

Based on eqs 4 and 11, we also calculated a hydrodynamic diameter of the species in solution, demonstrating that *d*_h_values (eq 11), perhaps unsurprisingly, are much larger in water than in ethanol. For example, *d*_h_ = 2.01 nm for PEG-Lipid I in ethanol and *d*_h_ = 15.9 nm in water (Table S2). The data were similar and consistent between the PEG-Lipid I and PEG-Lipid II (vide infra).

### Dynamic Light Scattering (DLS) and AUC Data

**Figure 3:**
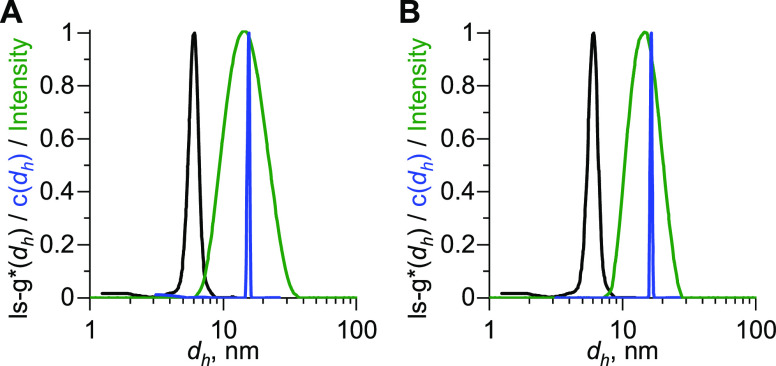
Normalized hydrodynamic size distributions in water derived from sedimentation analysis without considering effects of diffusion, *ls* – g*(*d*_h_) (black lines), and sedimentation–diffusion analysis considering translational friction properties, *c*(*d*_h_) (blue lines), as well as intensity-based hydrodynamic size distributions from DLS (green lines). (A) PEG-Lipid I and (B) PEG-Lipid II.

The derived *d*_h_ values from DLS are based on eq 1. For a monodisperse population of species, a cumulant analysis leads to intensity-based sizes, *d*_h, DLS_, accompanied with a polydispersity index (PDI). Figure S5 shows decay functions for PEG-Lipid I and PEG-Lipid II, while Figure 3A,B shows the respective intensity-based hydrodynamic size distributions in water. Decay functions (Figure S5) and *d*_h, DLS_ values (Figure 3 and Table S2) are very similar for the studied PEG-Lipids I and II. Figure 3A,B also show a comparison of the intensity-based distributions of hydrodynamic diameters from DLS, *d*_h, DLS_, with those based on sedimentation analysis, *ls* – g*(*d*_h_), and sedimentation–diffusion analysis, *c*(*d*_h_), by including the translational frictional ratios, *f*/*f*_sph_ (eq 11 and Table S2). The differential distributions of hydrodynamic diameters from AUC, *ls* – g*(*d*_h_) or *c*(*d*_h_), and resulting *d*_h,AUC_ values were derived as reported recently.^26^ The *z*-average *d*_h, DLS_ and average *d*_h,AUC_ values from sedimentation–diffusion analysis practically coincide (Table S2). The *c*(*d*_h_) distribution appears less disperse from AUC analysis than from DLS, which could be caused by (i) the diffusion-corrected differential distribution of sedimentation coefficients, *c*(*s*) (Figures 2C,D and S2C,D) and consequently hydrodynamic diameters, *c*(*d*_h_), (Figure 3) and (ii) a possible improper representation of the actual PEG-Lipid micelle size distribution from DLS data.^49^ Derived sizes without considering the translational frictional ratios, *f*/*f*_sph_, result in much smaller sedimentation-equivalent hydrodynamic sizes (see black solid lines in Figure 3).^36^*f*/*f*_sph_ values appear much larger in water when compared to the PEG-Lipid unimers in ethanol (Figures 2F and S2F). Overall, it appears that the water solution contains well-defined colloidal species of very similar hydrodynamic sizes by making use of AUC and DLS.

### Cryo-TEM

**Figure 4:**
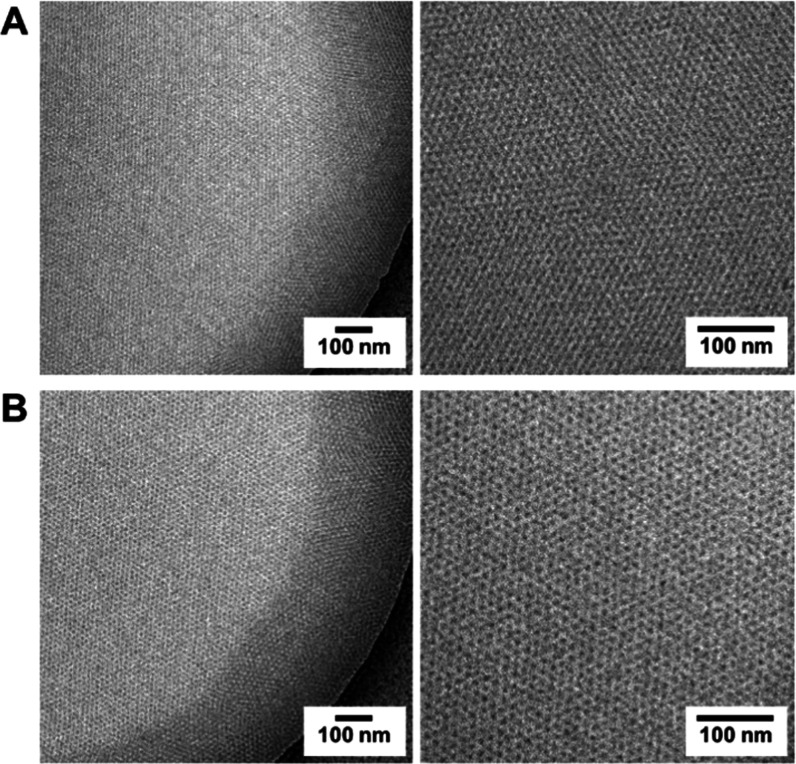
Cryo-TEM images from preparations of (A) PEG-Lipid I and (B) PEG-Lipid II in water.

Figure 4 shows cryo-TEM images of the PEG-Lipid I (Figure 4A) and PEG-Lipid II (Figure 4B) at different magnifications from dissolutions in water. The uniform character of the formed spherical micelles is apparent from the perfect hexagonal arrangement in the vitrified ice films. The resulting hexagonal form of the arrangement in the samples is moreover observable in the fast Fourier transform (FFT) representations (Figure S6). Such strictly organized micellar arrangements are a result of micellar repulsion^50^ and a thickness gradient in the concave ice-film formation in the holes of the TEM support grid.^51^ Domain boundaries between well-defined areas of hexagonal arrangement are also visible. Even though the internal architecture of the micelles cannot be revealed by cryo-TEM due to the low contrast of their outer shell, the strict hexagonal arrangement allows determining the diameter of an entire micelle. Micellar diameters were determined by analyzing line plots through arranged micelles (Figure S7). The sizes are averaged over at least 15 data points determined from several lines. The average diameter (measured as distance between two minima of the intensity,^52^Figure S7), was found to be *d*_cryo-TEM_ ≈ 15 nm for both PEG-Lipids (Table S2). Thus, obtained sizes, *d*_cryo-TEM_, are found to be very similar to those from DLS, *d*_h,DLS_, and AUC analysis, *d*_h,AUC_.

### Discussion of the Comprehensive Data

In this work, we report on the hydrodynamic characteristics of PEG-Lipids in two solvents differing substantially in solvent quality. Ethanol, as a less polar solvent compared to water, behaves as a better solvent, particularly for the hydrophobic part of the PEG-Lipids. Water, on the contrary, is a strongly polar solvent. It acts as a readily poor solvent for the lipid structure but a good solvent for the PEG chain. In this situation, hydrophobic lipids try to minimize contact with water, leading to the formation of micelles. The comprehensive data from different experimental studies in those solvents and the established values of [*η*] (eqs 6–8), [*s*] (eq 4), and [*D*] (eq 5) are compiled in Table S1. This table as well contains values of *A*_0_ (eq 9), possible to determine for any macromolecular and colloidal system.^12^ All primary hydrodynamic characteristics as well as derived parameters allow for the following observations: (i) The system studied in ethanol allows establishment of *A*_0_ values well in agreement to the scientific literature for PEG polymer unimers demonstrating also that the solution behavior and molar masses based on hydrodynamic analysis (eq 10) are physically consistent (Table S1);^7,11^ (ii) the polymer system in water shows *A*_0_ values below the theoretical limit of a solid impermeable sphere (*A*_0_ = 2.9 × 10^–10^ g cm^2^ K^–1^ mol^–1/3^)^12,40^ as seen in Table S1, which has been associated to permeable spheres and was recently observed for hyperbranched macromolecular systems as well as for dendrimers;^10,12,53−55^ and (iii) for the systems in water, the data for diffusion and/or hydrodynamic sizes are practically coinciding when utilizing AUC and DLS (Figure 3), supported by sizes derived from direct imaging via cryo-TEM (Figures 4, S6, and S7) as seen in Table S2.

In addition, data on translational friction properties from sedimentation–diffusion analysis (*f*/*f*_sph_ (eq 12)) evidence the impact of hydration (*δ*_AUC_ (eq 13)). Independent data based on viscometric investigations ([*η*] (eq 14)) and hydration (*δ*_visco_ (eq 15)) support the observation from sedimentation–diffusion analysis, except that somehow lower values of hydration are obtained from viscometric investigations (Table S2). The micelles are uniform in shape as indicated by the perfect hexagonal arrangement in cryo-TEM (Figures 4, S6, and S7). The micelles are, thereby, characterized by a high level of hydration, averaging to as much as 4–5 g water per g of polymer material constituting the micelle (Table S2). For example, proteins, though controversially discussed in the scientific literature in instances, have average levels of hydration typically being *δ* = 0.36 g/g.^56,57^ Hyperbranched poly(glycerol)s (HPGs) have much larger values of hydration, averaging to *δ* = 1.7 g/g, that have been explained by the solvent-permeable hyperbranched structure, i.e., water partly being present in the internal polymer architecture.^10^ In the present case, values of hydration are more than twice as large than those of HPGs underpinning the large amounts of solvent, here water, contained in and associated to the micelles assembled from about 120 individual polymer chains (Table S2).

## Conclusions

Our study shows that the primary hydrodynamic data for the here presented PEG-Lipid systems can be used to quantitatively derive physically consistent metrics. We found that dissolution of the materials in ethanol led to unimolecular dissolved compact polymer chains, while dissolution in water resulted in the formation of micelles. Metrics such as molar masses, sizes, aggregation number, and hydration of the objects in solution allow the quantitative study of those systems. Notably, the hydration determined from intrinsic viscosity data agrees with translational friction data from radial- and time-resolved sedimentation velocity AUC recordings. Analyses by DLS and cryo-TEM fully support the data extracted from sedimentation velocity AUC experiments, particular by the verification of the uniform micelle size leading to a hexagonal arrangement clearly discernable from cryo-TEM data. With those data at hand, we are able to additionally address hydrodynamic characterization of related polymer systems in the quest for the replacement of PEGs and also anisotropic micelle systems in the future. This is because translational frictional ratios are inherently influenced by hydration and shape anisotropy. Those are difficult to distinguish by modeling sedimentation velocity data alone. The understanding of those systems for nanomedical translative applications, where shape anisotropy, in addition to hydration, plays a pivotal role for function can lead to the establishment of better structure–property relationships.

## Supporting Information Available

Additional experimental results and data evaluation from densimetry, sedimentation velocity AUC experiments, decay functions from DLS, cryo-TEM image analysis, and compiled hydrodynamic characteristics and properties (PDF)
