# Supplementary material for: PEG-Lipids: Quantitative Study of Unimers and Aggregates Thereof by the Methods of Molecular Hydrodynamics
Source: Anal Chem. 2023 Jul 7;95(28):10795–802. doi: 10.1021/acs.analchem.3c01999 (PMC10357408; doi:10.1021/acs.analchem.3c01999)
Supplement: Supplementary file 1 — ac3c01999_si_001.pdf [file ac3c01999_si_001.pdf]

Supporting Information

PEG-Lipids: Quantitative Study of Unimers and Aggregates Thereof by the Methods of Molecular Hydrodynamics

Ilya Anufriev[a,b], Stephanie Hoeppener[a,b], Ivo Nischang*[,a,b]

[a]Laboratory of Organic and Macromolecular Chemistry (IOMC), Friedrich Schiller University Jena, Humboldtstr. 10, 07743 Jena, Germany

[b]Jena Center for Soft Matter, Friedrich Schiller University Jena, Philosophenweg 7, 07743 Jena, Germany

*Corresponding Author

Ivo Nischang – Laboratory of Organic and Macromolecular Chemistry (IOMC) and Jena Center for Soft Matter (JCSM), Friedrich Schiller University Jena, 07743 Jena, Germany; orcid.org/0000-0001-6182-5215; Phone: +49-3641-948-569; Email: ivo.nischang@uni-jena.de

Table of content

1. Supporting Figures S1 to S7 .... S2

2. Supporting Tables S1-S2 .... S9

1. Supporting Figures S1 to S7

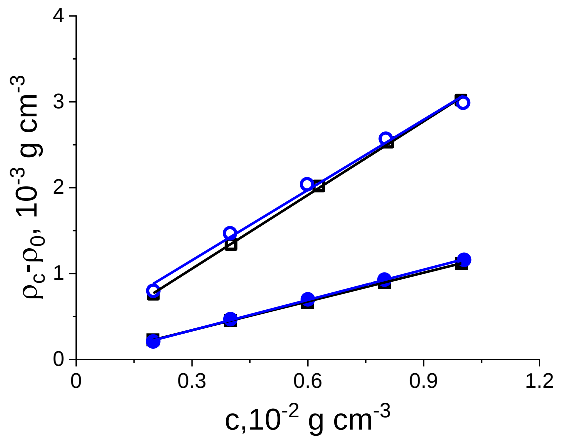

Figure S1. Dependence of $(\rho_c - \rho_0)$ on the concentration of PEG-Lipid I (black symbols and fit) and II (blue symbols and fit) in solvents ethanol (open symbols) and water (closed symbols).

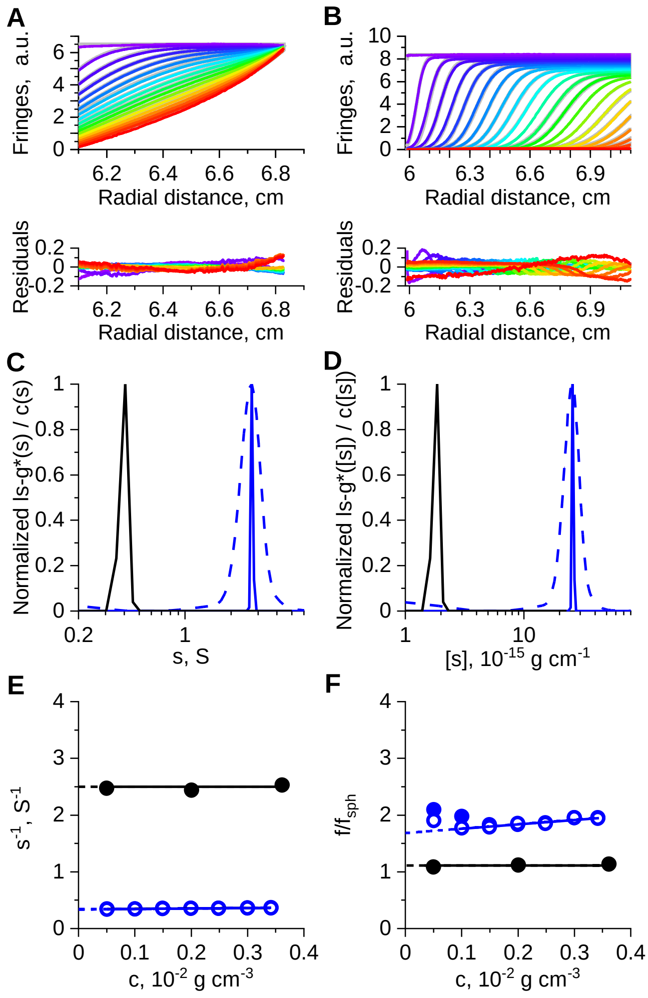

Figure S2. Time-resolved radial distance scans (gray symbols) and numerical results from sedimentation-diffusion analysis, $c(s)$, (colored lines) of PEG-Lipid II in (A) ethanol and (B) water at a concentration of $c = 2\ mg\ mL^{-1}$. (C) Differential distributions of sedimentation coefficients from sedimentation-diffusion analysis, $c(s)$ (solid black line in solvent ethanol, solid blue line in solvent water) and without considering effects of diffusion, $ls - g^{*}(s)$ (dashed blue line in solvent water). (D) Differential distributions of intrinsic sedimentation coefficients, $c([s])$ and $ls - g^{*}([s])$, based on (C) and derived on the basis of eq. 4. Concentration dependence of (E) signal (weight) average inverse sedimentation coefficients, s, and (F) weight-average translational frictional ratios, f/f_{sph}. Black symbols and lines in (E) and (F) refer to solvent ethanol with averages shown. Blue symbols and lines refer to solvent water with linear fits and extrapolation to infinite dilution. Open blue symbols refer to stability experiments.

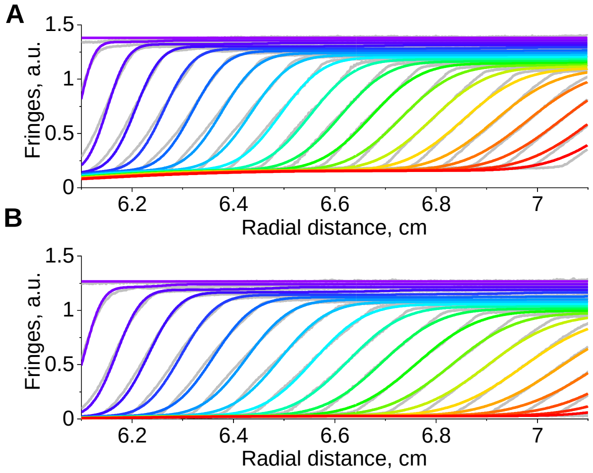

Figure S3. Time-resolved radial distance scans (gray symbols) and numerical results from sedimentation-diffusion analysis, $c(s)$, (colored lines) at the lowest investigated concentration ($c = 0.5\ mg\ mL^{-1}$) for (A) PEG-Lipid I and (B) PEG-Lipid II.

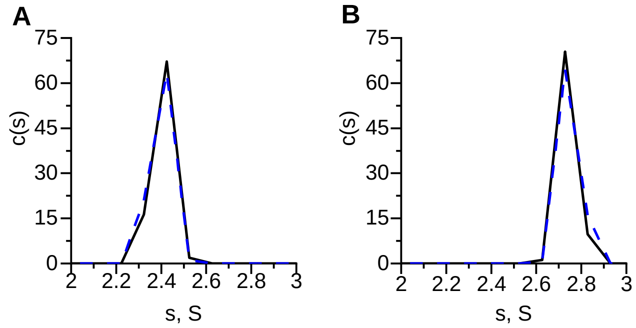

Figure S4. Example differential distributions of sedimentation coefficients from sedimentation-diffusion analysis, $c(s)$, with the original run and analysis (solid black lines) and the run and analysis after shaking the centrifuge cells (dashed blue lines) in water for (A) PEG-Lipid I and (B) PEG-Lipid II. Data are shown for a concentration of $c = 3.8 \ mg \ mL^{-1}$ for PEG-Lipid I and $c = 3.4 \ mg \ mL^{-1}$ for PEG-Lipid II.

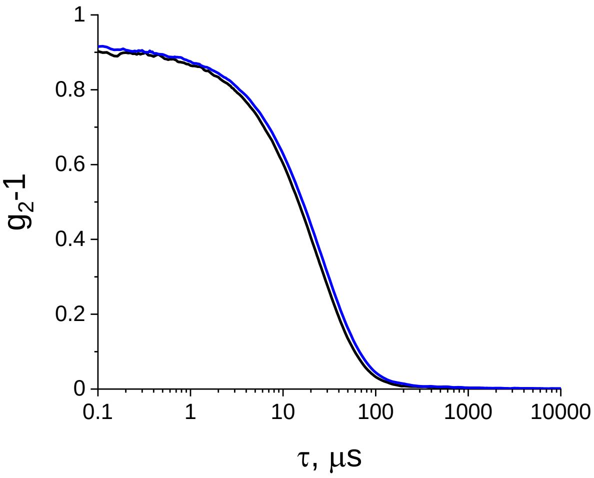

Figure S5. Decay functions of PEG-Lipid I (black trace) and II (blue trace) in water.

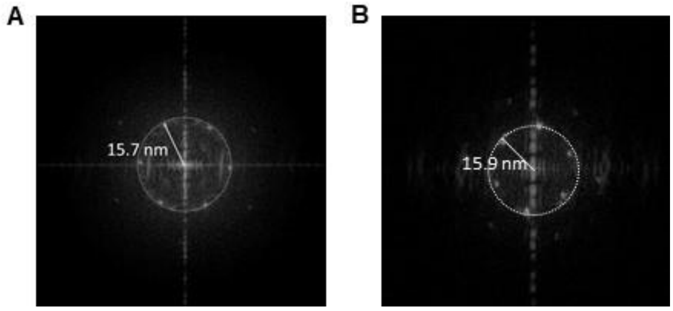

Figure S6. Fast Fourier Transform (FFT) representations from cryo-TEM images for (A) PEG-Lipid I and (B) PEG-Lipid II of preparations in water.

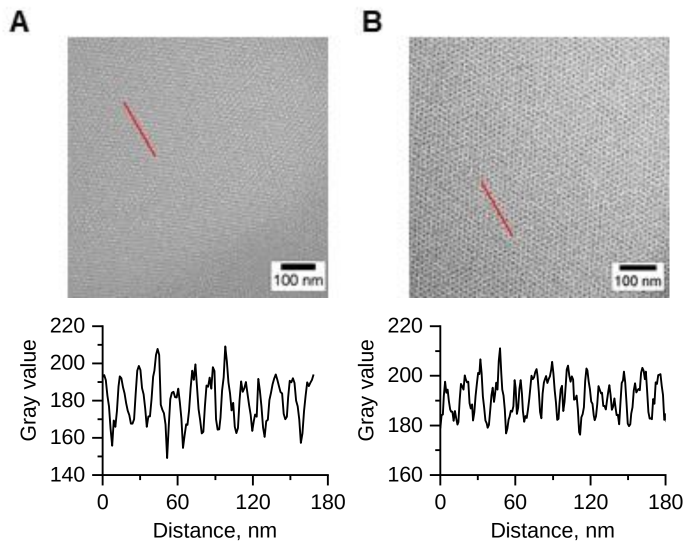

Figure S7. Example of pixel intensity line plots (of several used) showing maxima and minima for (A) PEG-Lipid I and (B) PEG-Lipid II preparations in water.

2. Supporting Tables S1-S2

Table S1. Hydrodynamic characteristics of PEG-Lipid systems in solvents ethanol and water.

$Sample$	[a]$[\eta]$, $cm^3 g^{\cdot}$	k_h	k_k	υ, $cm^3 g^{-1}$	s, S	$[s], 10^{-15}$ $g\ cm^{-1}$	f/f_{sph}	$[D], 10^{-11}$ $cm\ g\ s^2\ K^{-1}$	$M_{s.f}$, $kg\ mol^{-1}$	$A_0, 10^{-10}$ $g\ cm^2 K^{-1} mol^{-1/3}$
PEG-Lipid I (Ethanol)	6.7	0.69	0.05	0.90	0.38	1.7	1.18	6.99	2,0	3.55
PEG-Lipid II (Ethanol)	5.9	0.77	0.04	0.92	0.41	1.9	1.07	7.39	2,1	3.71
PEG-Lipid I (Water)	12.2	-	-	0.89	2.62	24.8	1.85	0.86	224,0	2.75
PEG-Lipid II (Water)	12.1	-	-	0.89	2.94	27.8	1.87	0.96	269,0	2.78

[a]Average values obtained from Huggins- and Kraemer.

Table S2. Hydrodynamic diameters and hydration values of the PEG-Lipids in water

$Sample$	$^a d_{h,AUC}$ nm	$d_{h,AUC}$ nm	$d_{h,DLS}$ nm	$d_{cryo-TEM}$ nm	N_{agg}	δ_{visco} g/g	δ_{AUC} g/g
PEG-Lipid I	2.01	15.9 ± 1.8	16.0 ± 7.3	15.0 ± 0.9	112	3.98	4.70
PEG-Lipid II	1.98	17.1 ± 1.0	16.5 ± 8.7	15.1 ± 2.3	128	3.96	4.90

[a]Values obtained in ethanol
